# Isolation, characterization and therapeutic evaluation of phage HHU1 against K2 *Klebsiella pneumoniae*

**DOI:** 10.3389/fcimb.2025.1668727

**Published:** 2025-09-30

**Authors:** Pengjun Han, Shuting Sun, Liwen Wen, Lei Yang, Enzhong Li, Yang Yang

**Affiliations:** ^1^ Department of Neurosurgery, Zhumadian Central Hospital, Affiliated Central Hospital of Huanghuai University, Zhumadian, China; ^2^ College of Medicine, Huanghuai University, Zhumadian, China; ^3^ College of Biological and Food Engineering, Huanghuai University, Zhumadian, China

**Keywords:** *Klebsiella pneumoniae*, phage HHU1, biological properties, phage therapy, drug-resistant

## Abstract

The prevalence of multidrug-resistant *Klebsiella pneumoniae (K. pneumoniae)* poses a severe threat to the global economy and public health, driving a resurgence of interest in phage therapy. Consequently, it is imperative to isolate lytic phages against *K. pneumoniae* with potent bactericidal activity. This study reports the isolation and characterization of the phage HHU1 against *K. pneumoniae* from hospital sewage, to evaluate its potential for phage therapy. Phage HHU1 has an icosahedral head and retractable tail, similar to members of the *Jedunavirus* genus. Host range tests revealed that phage HHU1 obligately lysed *K. pneumoniae* of the K2 serotype. Genome sequencing analysis showed that the genome of phage HHU1 was 47,779 bp in length, with a GC content of 49.2%, encoding 78 open reading frames, and lacked genes associated with lysogeny and virulence. The rapid adsorption (8 min), short latent period (10 min), and high burst size (approximately 134 PFU/cell) indicate robust replication kinetics of phage HHU1. Phage HHU1 remained stably active even after incubation for 6 h at pH 5.0-10.0 and temperature 4-40°C. In addition, phage HHU1 with different MOIs can completely inhibit the growth of drug-resistant *K. pneumoniae* within 8 h *in vitro* and significantly reduce biofilm formation of drug-resistant bacteria. Notably, treatment with high-dose phage HHU1 (MOI = 0.1 and 1) achieved 100% survival in *Galleria mellonella* larvae infected with drug-resistant *K. pneumoniae*. These findings demonstrate the potential of phage HHU1 as a promising therapeutic candidate against drug-resistant *K. pneumoniae* infections.

## 1 Introduction

*Klebsiella pneumoniae (K. pneumoniae)* is one of common Gram-negative bacillus and the most important opportunistic hospital pathogens (Wyres et al., 2020). Based on phenotypic and genotypic characteristics, *K. pneumoniae* can be classified into two types: classical *K. pneumoniae* (cKp) and hypervirulent *K. pneumoniae* (hvKp) (Pomakova et al., 2012). Notably, hvKp strains can cause community-acquired infections in immunocompetent individuals of any age group, leading to severe clinical manifestations including pyogenic liver abscesses, pneumonia, and central nervous system infections such as meningitis and brain abscesses (Ye et al., 2016; Russo and Marr, 2019; Lei et al., 2024). hvKp strains produce capsular polysaccharides that serve as critical virulence determinants, and the majority of hvKp isolates belong to the K1 and K2 capsular serotypes (Al-Busaidi et al., 2024), among which the K2 serotype isolates are more genetically diverse (Lai et al., 2019). The widespread use of antibiotics to treat infections caused by *K. pneumoniae* has led to the emergence and spread of bacterial resistance. In recent years, the resistance rate of hvKp to carbapenem antibiotics has increased significantly in China (Yang et al., 2022), and polymyxin-resistant hvKp have also appeared frequently (Jin et al., 2021). Given the escalating challenge of antibiotic treatment failures in hvKp infections, there is a critical imperative to develop novel therapeutic approaches.

Bacteriophages (phages) are naturally occurring bacterial viruses that can rapidly and selectively infect and lyse host bacteria (Górski et al., 2020). Phages have been used to treat bacterial infectious diseases since their discovery, but the increasing maturity and popularity of antibiotic treatments has led to a stunting of phage therapy (Salmond and Fineran, 2015). The escalating crisis of antimicrobial resistance in recent years has prompted a resurgence of scientific interest in phage therapy. Substantial evidence now supports both the efficacy and safety profile of phage therapy in mammalian models and clinical settings (Hung et al., 2011; Bao et al., 2020; Uyttebroek et al., 2022). Nevertheless, several challenges remain, including the narrow host range of individual phages, and the rapid emergence of bacterial resistance to a single phage (Torres-Barceló et al., 2022; Chung et al., 2023). The addition of a new phage to a therapeutic cocktail is a key strategy to address these limitations. It can broaden the spectrum of activity against a wider array of bacterial strains or species, mitigate the risk of resistance development through simultaneous targeting of different bacterial receptors or pathways, and enhance the overall efficacy of the treatment through synergistic effects (Yang et al., 2023; Chen et al., 2024; Kim et al., 2025). Since the potential success of phage therapy depends on having a large number of phages or so-called phage biobanks, the isolation and characterization of new phages targeting different types of drug-resistant bacteria is still necessary. In addition, analysis of the phage genome to ensure the absence of virulence and resistance genes is a necessary prerequisite for phage therapy.

As of June 2025, nineteen characterized lytic phages targeting K2-type hvKp (K2-hvKp) had been documented, based on a PubMed literature search. Among these, phages KlebP_265 (Yakubovskij et al., 2025), ΦFK1979 (Tang et al., 2023), vB_KpP_HS106 (Chen et al., 2023) and PSKP16 (Rahimi et al., 2023) showed relatively good lytic properties, providing a reserve resource for the treatment of K2-hvKp infections. However, the current repertoire of isolated phages remains critically insufficient relative to the clinical prevalence of K2-hvKp strains. There exists an urgent need to expand the phage resource bank through systematic environmental discovery, thereby diversifying therapeutic options for future clinical management of K2-hvKp infections.

In this study, the lytic phage HHU1 was isolated from hospital sewage samples using a K2 capsular serotype *K. pneumoniae* clinical isolate (strain 1301) as the host. Through comprehensive characterization of its biological properties and whole-genome sequence, coupled with assessment of its bactericidal efficacy against the host bacterium in both *in vitro* and *in vivo* models, phage HHU1can be suggested as a potentially therapeutic agent against K2-hvKp.

## 2 Materials and methods

### 2.1 Bacterial strains and culture conditions

All clinical isolates of *K. pneumoniae* strains with diverse capsular serotypes used in this study were obtained from hospital specimens and donated by Professor Yigang Tong (Beijing University of Chemical Technology). Strains were preserved in cryovials containing LB medium with 25-30% glycerol at -80°C. Strains cultured in Luria-Bertani (LB) broth at 37°C with shaking (220 rpm). Bacterial colony isolation and enumeration were performed on LB agar plates (1.5% w/v).

### 2.2 Phage isolation and purification

Hospital sewage samples were centrifuged at 12,000 × g for 10 min, and the supernatant was filtered through 0.45-μM filter unit (Pall, USA). Subsequently, 10 mL of filtrate was added to 100 mL of *K. pneumoniae* 1301 culture at mid-logarithmic phase (OD_600_ ≈ 0.6), followed by incubation with shaking (220 rpm) at 37°C for 8 h. The mixture was then centrifuged and filtered. The resulting filtrate underwent serial dilution with sterile water, and 100 μL aliquots of each dilution were mixed with 0.5 mL of mid-log phase bacterial culture for double-layer agar plaque assays (Han et al., 2024). After solidification, plates were inverted and incubated overnight at 37°C. Individual plaques were picked and transferred to 5 mL of mid-log phase bacterial culture. This enrichment cycle - comprising incubation, centrifugation, filtration, dilution, and co-cultivation - was repeated for three successive rounds to purify the isolated phages.

### 2.3 Electron microscopy

Phage HHU1 suspensions were concentrated via cesium chloride (CsCl) density gradient centrifugation (Han et al., 2022) and resuspended in 1 mL of sterile deionized water. A 5 μL aliquot of concentrated phage suspension was applied to each copper grid and incubated for 10 min at room temperature. Excess liquid was removed using filter paper wedges by capillary action from the grid edge. Grids were then blotted on fresh filter paper for 1 min. Subsequently, 10 μL of 2% uranyl acetate solution was applied to the grid for 90-second staining. Residual stain was removed with filter paper wedges, followed by air-drying on filter paper for 3 h in a desiccator. Finally, samples were examined using transmission electron microscopy (TEM) (JEM-1200EX, JEOL, Tokyo, Japan) at 80 kV.

### 2.4 Determination of the optimal multiplicity of infection

Phage concentrate (1×10^9^ PFU/mL) was subjected to serial dilutions. Aliquots (100 μL) from each dilution were mixed with 100 μL of bacterial suspension (1×10^8^ CFU/mL) according to multiplicity of infection (MOI) values of 0.0001, 0.001, 0.01, 0.1, 1, and 10. The mixtures were inoculated into 5 mL of liquid medium and incubated at 37°C with shaking for 12 h. After incubation, the cultures were centrifuged and filtered. Phage titers were determined using the soft agar overlay method (Li and Zhang, 2014). The MOI corresponding to the highest phage titer was identified as the optimal MOI.

### 2.5 Determination of adsorption curve and one-step growth curve

Adsorption experiment was carried out as previously described with some modifications (Han et al., 2023). Briefly, *K. pneumoniae* strain 1301 was inoculated into 100 mL of fresh LB liquid medium and cultured to the logarithmic growth phase. Subsequently, phage HHU1 was added and incubated statically at 37°C, and aliquots (1 mL) were collected at 0, 2, 4, 6, 8, 10, 12, 14, 16 min post-infection. Each sample was immediately centrifuged at 15,000 × g for 1 min at 4°C, and 100 μL of the supernatant was used to quantify free phage titer via the soft agar overlay method. The unadsorbed phage ratio at each time point was calculated relative to the 0-min baseline titer using the formula:


Unadsorbed ratio(%)=(Detection  titer/Baseline titer) ×100

A 2 mL of *K. pneumoniae* strain 1301 culture (1 × 10^7^ CFU/mL) was mixed with 2 mL of phage HHU1 suspension (1 × 10^6^ PFU/mL). The mixture was incubated stationary at room temperature for 10 min, followed by centrifugation at 12,000 × g for 2 min to pellet the bacterial cells. The pelleted cells were washed twice with sterile water and then resuspended in 20 mL of fresh LB broth. The suspension was incubated with shaking at 37°C for 90 min. During this incubation period, samples were collected at 10 min intervals starting from 0 min. Each sample was centrifuged at 12,000 × g for 2 min to harvest the supernatant. The supernatants collected at each time point were subjected to serial dilution, and the phage titer was determined using soft agar overlay method. This experiment was performed in triplicate. A one-step growth curve was constructed by plotting phage titer against infection time. The latent period and lysis period of phage HHU1 were determined from the curve, and the burst size was calculated according to the formula:


$$\begin{array}{c} \text{Burst size }\left(\text{PFU}/\text{cell}\right)\;=\;\left(\text{Final phage titer at plateau phase}\right)\\ /(\text{Initial number of infected host bacteria}) \end{array}$$

### 2.6 Host range analysis of the phage HHU1

The lytic activity of phage HHU1 against 33 K*. pneumoniae* clinical isolates and 16 other Gram-negative bacteria was evaluated using a spot test assay (Kim et al., 2020). Briefly, each bacterial strain was cultured to the logarithmic growth phase (OD_600_ ≈ 0.6). Subsequently, 0.5 mL of the bacterial culture was mixed with 5 mL of LB semi-solid medium (approximately 48°C) and evenly overlaid onto an LB agar plate. After solidification, 2 μL of phage HHU1 suspension (1×10^8^ PFU/mL) was spotted onto the center of each plate. The plates were then incubated at 37°C overnight. Finally, plaque formation was observed by examining the presence of clear zones on each plate.

### 2.7 Thermal and pH stability of the phage HHU1

Aliquots (900 μL) of fresh LB broth were dispensed into sterile 1.5 mL centrifuge tubes and incubated in a metal bath at 4, 20, 30, 40, 50, 60, and 70°C for 30 min. Following this, 100 μL of phage suspension (approximately 2×10^10^ PFU/mL) was added to each tube, and samples were incubated at their respective temperatures for 6 h and 12 h. After incubation, phage titers were determined using the soft agar overlay method to evaluate thermal stability. All experiments were performed in triplicate.

For pH stability assessment, LB liquid medium was adjusted to pH values ranging from 2.0 to 14.0 using 1 mol/L HCl and 1 mol/L NaOH. Subsequently, 100 μL of phage suspension (approximately 2×10^10^ PFU/mL) was added to each 1.5 mL centrifuge tubes containing the pH-adjusted LB broth. The mixtures were incubated at 30°C for 6 h and 12 h, respectively. Following incubation, phage titers were determined via the soft agar overlay method to evaluate phage stability. All experiments were performed in three independent replicates.

### 2.8 Genome extraction and sequencing

The genomic DNA of phage HHU1 was extracted using the Viral DNA Kit (Omega, Norcross, Georgia, USA) according to the manufacturer’s protocol. DNA libraries were prepared using the NEBNext Ultra™ II DNA Library Prep Kit for Illumina (New England Biolabs Ltd., Beijing, China). Following library preparation, sequencing was performed on the NovaSeq 6000 platform (Illumina, San Diego, USA) using the NovaSeq 6000 S4 Reagent Kit v1.5 (Illumina, San Diego, USA) with a paired-end 150 (PE150) sequencing strategy. The raw sequencing data were processed using Trimmomatic (v0.32) to filter low-quality reads (Bolger et al., 2014), and the filtered reads were assembled into the complete phage genome using SPAdes (v3.13.0) (Bankevich et al., 2012).

### 2.9 Phage genome annotation and bioinformatics analysis

The genome of phage HHU1 was analyzed using the RAST online server (Brettin et al., 2015) (https://rast.nmpdr.org/) to predict open reading frames (ORFs). Functional annotation of the encoded proteins was performed using the BLASTp tool (Basic Local Alignment Search Tool for proteins) available on the NCBI platform (https://www.ncbi.nlm.nih.gov/). HHpred against protein data bank (PDB) and Pfam database were used to predict more distant homologs (Söding et al., 2005). The annotated genome sequence was subsequently submitted to the NCBI database. tRNA-encoding genes within the phage genome were detected using tRNAscan-SE (Lowe and Chan, 2016) (http://lowelab.ucsc.edu/cgi-bin/tRNAscan-SE2.cgi). Putative virulence genes and pathogenicity-associated genes harbored by the phage genome were predicted using the Virulence Factor Database (VFDB) (Liu et al., 2022) (http://www.mgc.ac.cn/VFs/) and PathogenFinder (Cosentino et al., 2013) (http://cge.cbs.dtu.dk/services/PathogenFinder/), respectively. Genome visualization was conducted with the Proksee online tool (Grant et al., 2023) (https://proksee.ca/). For phylogenetic analysis, amino acid sequences exhibiting high homology to HHU1’s major capsid and terminase large subunit proteins were retrieved from NCBI. A phylogenetic tree was constructed using MEGA7 software (Kumar et al., 2016) with the ClustalW algorithm for sequence alignment and the Neighbor-Joining method for tree inference.

### 2.10 *In vitro* bactericidal activity test

Test was performed according to Łubowska et al. (2019) with minor modification. Briefly, an overnight culture of *K. pneumoniae* strain 1301 was diluted 1:100 (v/v) in fresh LB broth and incubated at 37°C with shaking until reaching the early logarithmic phase (OD_600_ ≈ 0.3). Phage suspensions were then added at MOI of 0.001, 0.01, 0.1, 1, and 10, followed by incubation at 37°C with shaking for 8 h. During the incubation period, samples were collected hourly to monitor bacterial growth via OD_600_ measurements using the Evolution One spectrophotometer (Thermo Fisher Scientific, Waltham, MA, USA). All the experiments were repeated three times.

### 2.11 Effect of phage HHU1 on biofilm formation

As previously described (Tabassum et al., 2018), the effect of phage HHU1 on biofilm formation by multidrug-resistant *K. pneumoniae* strain 1301 was assessed through co-incubation of phage and bacteria at 37°C. Strain 1301 was inoculated into Tryptic Soy Broth (TSB) medium and cultured with shaking until reaching an OD_600_ of 0.3. Subsequently, 100 μL of the bacterial suspension was aliquoted into a 96-well microtiter plate (Thermo Fisher Scientific, Waltham, MA, USA), followed by the addition of 100 μL of phage HHU1 suspensions at final MOI values of 0.001, 0.01, 0.1, and 1. An equal volume of sterile phosphate-buffered saline (PBS) was used as a negative control instead of phage suspension. The mixture was thoroughly mixed and incubated at 37 °C for 48 h (three biological replicates per group). After incubation, biofilm formation was quantified using the crystal violet staining assay (Ding et al., 2023). Finally, bound crystal violet was solubilized with 200 μL of 95% ethanol, and the absorbance was measured at 590 nm using the Multiskan SkyHigh Microplate Spectrophotometer (Thermo Fisher Scientific, Waltham, MA, USA).

To quantify the viable cells within the biofilm, phage HHU1 was mixed with the host strain 1301 at different MOIs and co-incubated statically for 48 h as described above. After incubation, the planktonic cells were removed, and the biofilm was gently washed twice with 200 μL of sterile PBS. The biofilm was then scraped and resuspended in PBS, followed by sonication in a 96-well plate for 10 min to disrupt the biofilm structure and release the bacterial cells. Subsequently, 100 µL droplets of tenfold serial dilutions ((10^−1^ to 10^−6^) of the cell suspension were spotted onto LB plates and evenly spread across the surface using a sterile spreader to ensure uniform distribution. The plates were incubated at 37°C for 16 h, and the colonies were enumerated to evaluate the antibacterial efficacy of phage HHU1 against the biofilm.

### 2.12 *Galleria mellonella in vivo* infection model and phage therapy

*Galleria mellonella* (*G. mellonella*) larvae have been introduced as an alternative model to evaluate the efficacy of phage therapy (Tsai et al., 2016). In this study, *G. mellonella* larvae were obtained from Huiyude Biotech Company (Tianjin, China). To establish the infection model, the pathogenicity of strain 1301 toward *G. mellonella* was evaluated. Fifty larvae (weighing 250–300 mg each) were randomly assigned to five experimental groups (n=10 per group). Each group was injected with 5 µL of the following solutions: PBS (negative control), or strain 1301 suspensions at concentrations of 5×10^6^ CFU/mL, 1×10^7^ CFU/mL, 2×10^7^ CFU/mL, or 4×10^7^ CFU/mL. The larvae were then maintained at 37 °C in the dark for five days. Larval survival was recorded every 12 h, and individuals exhibiting no movement or melanization were considered dead. The median lethal dose (LD_50_) was calculated based on the infection model.

To evaluate phage therapy efficacy, *G. mellonella* larvae were administered bacterial and phage inoculations in a staged approach. Sixty larvae were randomly allocated into six groups (n = 10 per group) for different treatments. Group I: Infected with 5 µL of strain 1301 (1×10^7^ CFU/mL) at time 0, followed by 5 µL of PBS at 2 h post-infection (hpi). Group II: Administered 5 µL of PBS at time 0, followed by 5 µL of phage HHU1 (1×10^8^ PFU/mL) at 2 hpi. Groups III–VI: Infected with 5 µL of strain 1301 (1×10^7^ CFU/mL) at time 0, followed by 5 µL of phage HHU1 at 2 hpi with the following titers, Group III (1×10^4^ PFU/mL), Group IV (1×10^5^ PFU/mL), Group V (1×10^6^ PFU/mL), and Group VI (1×10^7^ PFU/mL). Survival rates were monitored and recorded as described above.

### 2.13 Statistical analysis

GraphPad Prism 8.0.1 (GrapPad Software, San Diego, CA, USA) was used to plot the data. One-way analysis of variance (ANOVA) was used to evaluate the difference between the experimental and control groups. A p-value<0.05 was considered statistically significant.

## 3 Results

### 3.1 Isolation, purification, and electron microscopic morphology of phage HHU1

**Figure 1:**
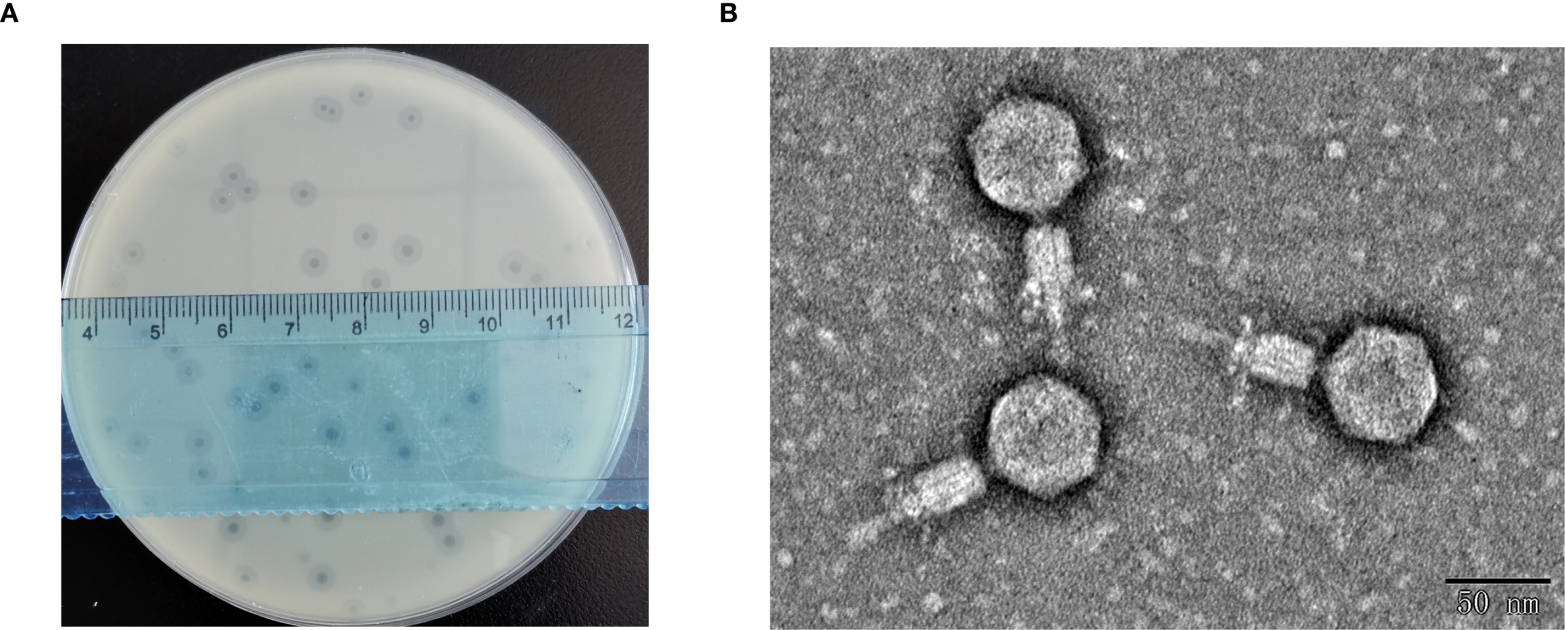
Morphological characterization of phage HHU1. **(A)** Lytic plaques formed by phage HHU1 on a lawn of *K. pneumoniae* strain 1301 following 8 h incubation at 37°C. **(B)** Transmission electron micrograph of phage HHU1. Scale bar is 50 nm.

Using *K. pneumoniae* strain 1301 as the host bacterium, a lytic phage designated HHU1 was isolated from sewage collected at Zhumadian Central Hospital. Following three rounds of purification, the phage formed uniform lytic plaques on double-layer agar plates containing the strain 1301. The plaques exhibited a mean diameter of 3.8 ± 0.24 mm, characterized by a clear center surrounded by a translucent halo (**Figure 1A**). The presence of a halo suggests the phage’s potential ability to produce depolymerase for degrading bacterial capsular polysaccharides (Zhao et al., 2024).

TEM revealed that phage HHU1 possesses an icosahedral head and a contractile tail. The head diameter was measured as 54.62 ± 2.37 nm (n=10), while the tail diameter was 66.75 ± 3.18 nm (n=10) (**Figure 1B**). Morphologically, phage HHU1 belongs to the *Myoviridae* family and exhibits structural similarities to the well-characterized phage T4.

### 3.2 Replication characteristics of phage HHU1

**Figure 2:**
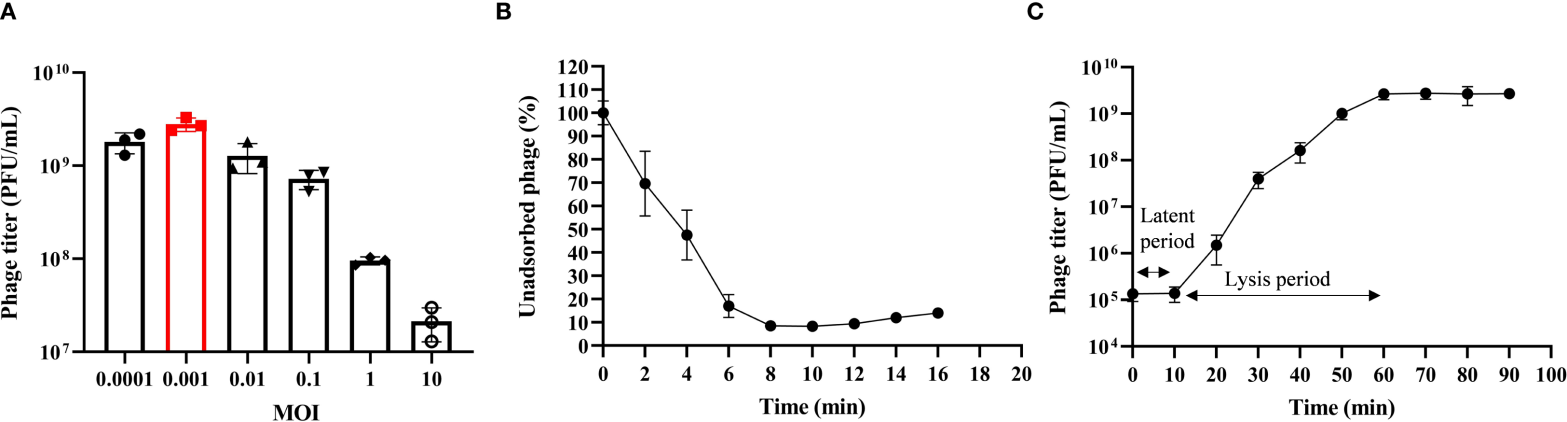
Biological characteristics of phage HHU1. **(A)** Multiplicity of infection determination for phage HHU1. **(B)** Adsorption curve of phage HHU1. **(C)** One-step growth curve of phage HHU1. Data are shown as the mean ± SD.

Phage HHU1 was co-incubated with *K. pneumoniae* strain 1301 at different MOIs. When infecting at an MOI of 0.001, phage HHU1 yielded the highest progeny production following host lysis (**Figure 2A**). These findings indicate that 0.001 represents the optimal MOI for maximizing progeny release during HHU1 replication.

Adsorption is the initial step for phages to infect bacteria. The adsorption kinetics of phage HHU1 were analyzed to determine its binding capacity to the host bacterium. As shown in **Figure 2B**, approximately 50% of HHU1 phages adsorbed onto the host surface within 4 min of co-incubation. Following 8 min of incubation, over 90% of phages had completed adsorption, after which the adsorption rate plateaued.

The latent period, lysis period, and progeny phage yield during HHU1 replication in the host bacterium can be determined through one-step growth curve analysis. As shown in **Figure 2C**, the phage titer remained unchanged for the initial 10 min, followed by a progressive increase until plateauing at 60 min post-infection. This indicates a latent period of 10 min and a lysis period of 50 min for phage HHU1. Based on the initial bacterial count (approximately 2×10^7^ CFU/mL) and the phage titer at the plateau phase (mean 2.68×10^9^ PFU/mL), the burst size of phage HHU1 was calculated as 134.17 ± 33.81 PFU/cell using the methodology described in the Materials.

### 3.3 Host range of phage HHU1

The host range of phage HHU1 was determined using 33 clinically isolated *K. pneumoniae* strain strains. The results revealed that HHU1 exhibited lytic activity against 7 of the 33 tested strains, all of which belonged to the K2 serotype (**Table 1**). It is worth noting that these seven strains exhibited different levels of drug resistance (**Supplementary Table S1**). Unfortunately, phage HHU1 did not exhibit lytic activity against *K. pneumoniae* strains of other capsular types, nor did it lyse common hospital-acquired Gram-negative bacteria (**Supplementary Table S2**), underscoring its narrow and specific lytic host range.

**Table 1:** Lytic activity of phage HHU1 against clinical isolates.

Species	Strains	ST type	Capsular type	Susceptibility	Origin
*Klebsiella pneumoniae*	1301	ST65	K2	+	307 Hospital
	1307	ST65	K2	+	307 Hospital
	CH-03	ST65	K2	+	Central Hospital
	CH-05	ST65	K2	+	Central Hospital
	CH-71	ST86	K2	+	Central Hospital
	CH-113	ST86	K2	+	Central Hospital
	CH-188	ST86	K2	+	Central Hospital
	K7	ST23	K1	–	Aviation General Hospital
	064	ST23	K1	–	Aviation General Hospital
	081	ST23	K1	–	Aviation General Hospital
	2024	ST23	K1	–	Aviation General Hospital
	2752	ST23	K1	–	Aviation General Hospital
	2755	ST23	K1	–	Aviation General Hospital
	S-2007	ST23	K1	–	Aviation General Hospital
	1241	ST11	K15	–	307 Hospital
	1291	ST11	K27	–	307 Hospital
	1300	ST11	K16	–	307 Hospital
	2004	ST11	K47	–	Aviation General Hospital
	2005	ST11	K25	–	Aviation General Hospital
	2006	ST11	K47	–	Aviation General Hospital
	2008	ST11	K64	–	Aviation General Hospital
	2009	ST11	K64	–	Aviation General Hospital
	2012	ST11	K25	–	Aviation General Hospital
	2015	ST11	K47	–	Aviation General Hospital
	2022	ST11	K47	–	Aviation General Hospital
	2026	ST11	K64	–	Aviation General Hospital
	2086	ST11	K64	–	Aviation General Hospital
	2002	ST15	K19	–	Aviation General Hospital
	2003	ST15	K19	–	Aviation General Hospital
	2013	ST15	K19	–	Aviation General Hospital
	2017	ST15	K19	–	Aviation General Hospital
	2027	ST15	K19	–	Aviation General Hospital
	2068	ST15	K19	–	Aviation General Hospital

Symbols: “+” denotes the formation of transparent plaques following co-culture of phage HHU1 with the test bacterium; “-” indicates no plaque formation.

### 3.4 Thermal and pH stability of phage HHU1

**Figure 3:**
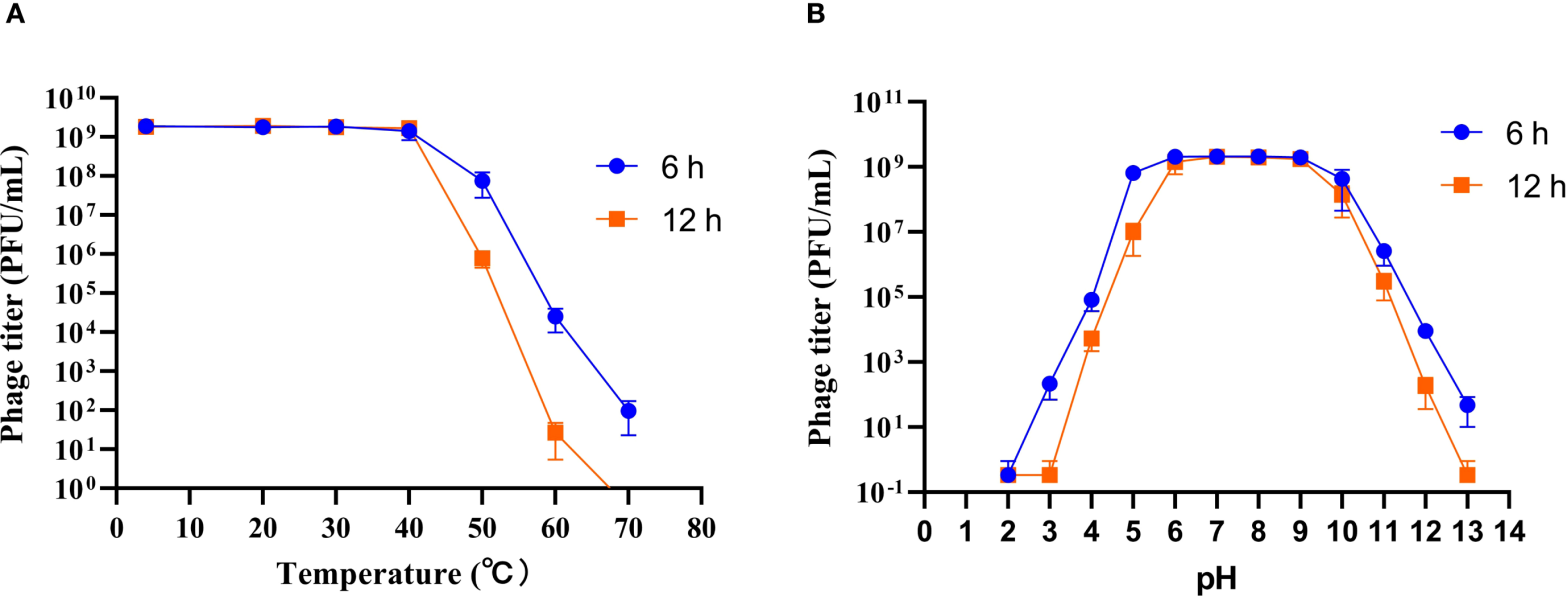
Stability of phage HHU1 under different temperature **(A)** and pH **(B)** conditions. Data are shown as the mean ± SD.

Temperature and pH are critical factors influencing the storage and application of phage. To evaluate these parameters, the stability of phage HHU1 was assessed under various temperature and pH conditions. As shown in **Figure 3A**, the titer of phage HHU1 remained virtually unchanged following 6 h or even 12 h of incubation at temperatures ranging from 4°C to 40°C. However, viability declined progressively at elevated temperatures, with complete inactivation occurring after 12 h at 70°C. These findings suggest that phage HHU1 maintains stability under ambient temperature conditions.

Regarding pH stability, phage titers remained essentially unchanged (≤0.5-log reduction) across pH 5–10 during 6 h incubations (**Figure 3B**). Exposure to acidic conditions (pH ≤4.0) induced significant titer reductions, culminating in complete inactivation at pH 3.0 following 12 h of incubation. Under alkaline conditions, a pH-dependent decrease in viability was observed, and total inactivation at pH 13.0 after 12 h. Collectively, these results demonstrate that phage HHU1 maintains exceptional stability across a broad pH range (5.0-10.0).

### 3.5 Genomic characterization of phage HHU1

Whole-genome sequencing and analysis of phage HHU1 revealed a double-stranded DNA genome of 47,779 bp with a GC content of 49.2%. The genome sequence was deposited in the NCBI GenBank database under the accession number PQ438797.1. Annotation of the HHU1 genome using bioinformatic software revealed a total of 72 open reading frames (ORFs). Among these, 26 ORFs demonstrated homology to functionally annotated proteins in the NCBI database, while the remaining ORFs showed homology to hypothetical proteins (**Table 2**). Notably, no known antibiotic resistance genes, virulence factor genes, or tRNA genes were identified within the HHU1 genome.

**Table 2:** Genomic annotation of phage HHU1.

ORFs	Strand	Start	Stop	Length (AA)	Predicted protein function	BLASTp result	Query cover	E-values	Identity	Accession
ORF1	+	384	965	193	hypothetical protein	*Klebsiella* phage vB_KpnM_JustaPhage	99%	3e-140	99.48%	YP_010684076.1
ORF2	–	1150	1001	49	hypothetical protein	*Klebsiella* phage RCIP0093	100%	2e-24	89.80%	WPJ55596.1
ORF3	–	2271	1147	374	hypothetical protein	*Klebsiella* phage RCIP0047	100%	0.0	97.59%	WPJ52268.1
ORF4	–	2567	2271	98	hypothetical protein	*Klebsiella* phage RCIP0063	100%	5e-64	98.98%	WPJ53524.1
ORF5	–	2755	2570	61	hypothetical protein	*Klebsiella* phage RCIP0054	100%	6e-35	98.36%	WPJ52911.1
ORF6	–	2916	2743	57	hypothetical protein	*Klebsiella* phage pKp383	100%	2e-32	98.25%	YP_010684176.1
ORF7	–	3128	2913	71	hypothetical protein	*Klebsiella* phage vB_KpnM_KpV52	100%	3e-42	95.77%	YP_009597597.1
ORF8	–	5581	3224	785	DNA primase	*Klebsiella* phage BUCT_47333	100%	0.0	98.22%	YP_010683798.1
ORF9	–	5882	5604	92	transcriptional regulator	*Klebsiella* phage vB_KpnM_JustaPhage	100%	6e-58	98.91%	YP_010684068.1
ORF10	–	6406	5945	153	hypothetical protein	*Klebsiella* phage vB_KleM_KB2	100%	2e-107	100.00%	YP_010684863.1
ORF11	–	6873	6403	156	hypothetical protein	*Klebsiella* phage RCIP0063	100%	3e-111	100.00%	WPJ53517.1
ORF12	–	8578	6908	556	helicase	*Klebsiella* phage vB_KpnM_KpV52	100%	0.0	99.82%	YP_009597592.1
ORF13	–	8789	8592	65	hypothetical protein	*Klebsiella* phage vB_KpnM_KpV52	100%	1e-40	100.00%	YP_009597591.1
ORF14	–	9012	8782	76	hypothetical protein	*Klebsiella* phage vB_KpnM_JustaPhage	100%	3e-47	100.00%	YP_010684062.1
ORF15	–	9186	9016	56	hypothetical protein	*Klebsiella* phage VLCpiM12a	100%	7e-31	98.21%	YP_010683818.1
ORF16	–	9410	9183	75	hypothetical protein	*Klebsiella* phage vB_KpnM_JustaPhage	100%	2e-48	98.67%	YP_010684060.1
ORF17	+	9551	10534	327	hypothetical protein	*Klebsiella* phage RCIP0063	100%	0.0	99.69%	WPJ53511.1
ORF18	+	10540	11664	374	exonuclease	*Klebsiella* phage vB_KpnM_JustaPhage	100%	0.0	98.66%	YP_010684058.1
ORF19	+	11667	11792	41	hypothetical protein	*Klebsiella* phage phage RCIP0030	100%	3e-18	100.00%	WPJ51019.1
ORF20	+	11877	12152	91	hypothetical protein	*Klebsiella* phage phage RCIP0030	100%	8e-58	100.00%	WPJ51018.1
ORF21	+	12232	13044	270	hypothetical protein	*Klebsiella* phage RCIP0063	100%	0.0	100.00%	WPJ53507.1
ORF22	+	13095	13214	39	hypothetical protein	*Klebsiella* phage	100%	9e-18	100.00%	WPJ53506.1
ORF23	+	13262	15436	724	DNA polymerase	*Klebsiella* phage MEW1	100%	0.0	95.44%	YP_010684111.1
ORF24	+	15433	15774	113	hypothetical protein	*Klebsiella* phage vB_KpnM_SCNJ1-Y	100%	4e-77	99.12%	WIL01649.1
ORF25	–	16036	15791	81	hypothetical protein	*Klebsiella* phage vB_KpnM_JustaPhage	100%	1e-48	98.77%	YP_010684049.1
ORF26	–	16314	16087	75	hypothetical protein	*Klebsiella* phage RCIP0045	100%	4e-47	100.00%	WPJ52121.1
ORF27	–	16629	16513	38	hypothetical protein	*Klebsiella* phage RCIP0063	100%	8e-16	100.00%	WPJ53501.1
ORF28	+	16684	17241	185	endolysin	*Klebsiella* phage KpTRp1	100%	7e-134	99.46%	XDG30680.1
ORF29	+	17241	17555	104	Rz-like spanin	*Klebsiella* phage vB_KpnM_FZ14	100%	1e-66	100.00%	YP_010684769.1
ORF30	+	17552	17788	78	hypothetical protein	*Klebsiella* phage RCIP0033	100%	3e-48	96.15%	WPJ51408.1
ORF31	–	18676	17783	297	tail fiber protein	*Klebsiella* phage KpTRp1	100%	0.0	98.99%	XDG30677.1
ORF32	–	19396	18680	238	structural protein	*Klebsiella* phage vB_KpnM_KpV52	98%	5e-170	98.72%	YP_009597572.1
ORF33	–	19670	19497	57	hypothetical protein	*Klebsiella* phage RCIP0063	100%	2e-31	98.25%	WPJ53495.1
ORF34	–	21687	19681	668	tail protein	*Klebsiella* phage VLCpiS13b	100%	0.0	99.10%	YP_009597570.1
ORF35	–	22892	21684	402	baseplate wedge subunit	*Klebsiella* phage vB_KpnM_JustaPhage	100%	0.0	100.00%	YP_010684039.1
ORF36	–	23232	22885	115	hypothetical protein	*Klebsiella* phage RCIP0030	100%	6e-75	99.13%	WPJ51001.1
ORF37	–	23906	23232	224	baseplate spike	*Klebsiella* phage vB_KleM_KB2	100%	2e-163	99.55%	YP_010684833.1
ORF38	–	24842	24192	216	baseplate protein	*Klebsiella* phage KpTRp1	100%	2e-152	99.54%	XDG30671.1
ORF39	–	26263	24839	474	baseplate hub	*Klebsiella* phage vB_KpnM_KpV52	100%	0.0	98.52%	YP_009597565.1
ORF40	–	27717	26266	483	tail length tape measure protein	*Klebsiella* phage 1611E-K2-1	100%	0.0	96.27%	YP_010684662.1
ORF41	–	28013	27717	98	hypothetical protein	*Klebsiella* phage vB_KpnM_KpVB3	100%	2e-64	96.94%	WQZ01381.1
ORF42	–	28161	27991	56	hypothetical protein	*Klebsiella* phage vB_KpnM_KpV52	100%	2e-34	100.00%	YP_009597562.1
ORF43	–	28574	28200	124	hypothetical protein	*Klebsiella* phage vB_KpnM_SCNJ1-Y	100%	2e-84	99.19%	WIL01631.1
ORF44	+	28701	28898	65	hypothetical protein	*Klebsiella* phage Geezett	100%	9e-36	93.85%	YP_010683917.1
ORF45	+	28895	29167	90	hypothetical protein	*Klebsiella* phage vB_KpnM_JustaPhage	100%	7e-61	98.89%	YP_010684029.1
ORF46	+	29164	29289	41	hypothetical protein	*Klebsiella* phage vB_KpnM_JustaPhage	100%	2e-21	100.00%	YP_010684028.1
ORF47	+	29289	29459	56	hypothetical protein	*Klebsiella* pneumoniae	100%	4e-31	100.00%	WP_243202410.1
ORF48	+	29459	29611	50	hypothetical protein	*Klebsiella* phage vB_KpnM_JustaPhage	100%	6e-28	100.00%	YP_010684026.1
ORF49	+	29608	30036	142	hypothetical protein	*Klebsiella* phage vB_KleM_KB2	100%	1e-58	69.93%	YP_010684912.1
ORF50	+	30153	30449	98	hypothetical protein	*Klebsiella* phage RCIP0113	100%	6e-63	97.96%	WPJ57023.1
ORF51	+	30532	31212	226	hypothetical protein	*Klebsiella* phage RCIP0015	100%	6e-157	94.69%	WPJ49148.1
ORF52	–	31667	31251	138	hypothetical protein	*Klebsiella* phage phiYH65	100%	4e-92	98.55%	XDG19024.1
ORF53	–	32814	31681	337	tail sheath	*Klebsiella* phage vB_KpnM_JustaPhage	100%	0.0	99.73%	YP_010684023.1
ORF54	–	33438	32857	193	hypothetical protein	*Klebsiella* phage RCIP0063	100%	6e-140	98.96%	YP_010685722.1
ORF55	–	33778	33425	117	hypothetical protein	*Klebsiella* pneumoniae	100%	5e-80	100.00%	WP_193852692.1
ORF56	–	34278	33775	167	tail completion or Neck1 protein	*Klebsiella* phage vB_KpnM_FZ14	100%	1e-114	96.41%	YP_010684824.1
ORF57	+	35029	35667	212	hypothetical protein	*Klebsiella* phage vB_KpnM_KpV52	100%	5e-153	99.53%	YP_009597552.1
ORF58	+	35747	35878	43	hypothetical protein	*Klebsiella* phagevB_KpnM_FZ14	100%	2e-22	100.00%	YP_010684817.1
ORF59	–	36406	35903	167	hypothetical protein	*Klebsiella* phage phiYH65	100%	1e-89	94.61%	XDG19031.1
ORF60	–	36960	36541	139	hypothetical protein	*Klebsiella* phage vB_KleM_KB2	100%	1e-99	99.28%	QNI20530.1
ORF61	–	37296	36979	105	hypothetical protein	*Klebsiella* phage RCIP0030	100%	2e-65	95.24%	WPJ50982.1
ORF62	–	38774	37329	481	endonuclease-like protein	*Klebsiella* phage phiYH65	96%	0.0	94.62%	XDG19034.1
ORF63	–	39858	38824	344	major head protein	*Klebsiella* phage vB_KpnM_FZ14	100%	0.0	96.22%	YP_010684811.1
ORF64	–	40349	39861	162	virion structural protein	*Klebsiella* phage vB_KpnM_KpV79	100%	1e-99	93.83%	YP_009615286.1
ORF65	–	41455	40361	364	head maturation protease	*Klebsiella* phage vB_KpnM_FZ14	100%	0.0	99.18%	YP_010684809.1
ORF66	+	41532	41753	73	nucleotide kinase	*Klebsiella* phage vB_KpnM_KpV79	100%	4e-46	100.00%	YP_009615284.1
ORF67	+	41753	41938	61	hypothetical protein	*Klebsiella* phage RCIP0071	100%	5e-37	98.36%	WPJ52081.1
ORF68	+	41941	42072	43	hypothetical protein	*Klebsiella* phage vB_KpnM_KpV52	100%	4e-22	95.35%	YP_009597541.1
ORF69	+	42069	42257	62	hypothetical protein	*Klebsiella* phage VLCpiM12a	100%	7e-38	100.00%	YP_010683868.1
ORF70	+	42241	42441	66	hypothetical protein	*Klebsiella* sp. GG_Kp142	95%	5e-34	85.71%	WP_348907484.1
ORF71	+	42509	42715	68	hypothetical protein	*Klebsiella* phage VLCpiM12a	98%	2e-16	56.72%	YP_010683870.1
ORF72	+	42708	42860	50	hypothetical protein	*Klebsiella* phage RCIP0015	100%	7e-25	94.00%	WPJ49125.1
ORF73	+	42857	43057	66	hypothetical protein	*Klebsiella* phage VLCpiM12a	100%	5e-29	78.79%	YP_010683872.1
ORF74	–	43808	43047	253	minor head protein	*Klebsiella* phage vB_KpnM_FZ14	100%	0.0	98.81%	YP_010684802.1
ORF75	+	43936	44244	102	hypothetical protein	*Klebsiella* phage RCIP0030	100%	6e-66	96.08%	WPJ50968.1
ORF76	–	45633	44236	465	portal protein	*Klebsiella* phage vB_KpnM_JustaPhage	100%	0.0	98.51%	YP_010684000.1
ORF77	–	47084	45636	482	terminase large subunit	*Klebsiella* phage SBP	100%	0.0	99.38%	YP_010684543.1
ORF78	–	47532	47074	152	terminase small subunit	*Klebsiella* phage JD001	100%	1e-104	98.68%	YP_007392856.1

The symbol “+” denotes a positive strand, while “-” denotes a negative strand.

**Figure 4:**
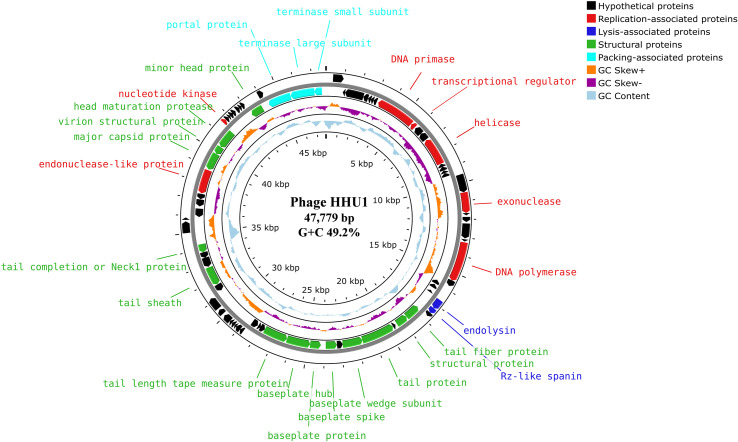
Genome map of phage HHU1 generated by Proksee. Circles from outermost to innermost correspond to (i) predicted ORFs on forward strand and (ii) reverse strand, (iii) GC skew +/– with the orange and purple histogram, (iv) cyan represents the (G+C) content, (v) genome length markers. The annotated protein functions are classified according to roles, and different colors represent different functional modules.

Based on the genomic positions of functional genes, the HHU1 genome exhibits a modular architecture, with functionally related genes predominantly clustered together. The functional protein within the phage HHU1 genome is visualized in **Figure 4**, revealing a genomic architecture primarily organized into four functional modules: phage lysis, packaging, replication and structural proteins.

Given that phage HHU1 forms plaques with halos on double-layer agar plates containing its host bacterium, it was hypothesized that the phage genome harbors a gene encoding a depolymerase. To investigate this, all proteins encoded in the genome of phage HHU1 were analyzed using DePolymerase Predictor (DePP), a tool designed for targeted identification of phage depolymerases (Magill and Skvortsov, 2023). The results indicated that the protein encoded by ORF34 (tail protein) exhibited the highest probability values (**Supplementary Table S3**). Subsequent alignment against PDB revealed that this protein shares 99.68% similarity with the established depolymerase KP32gp38 from *Klebsiella* phage KP32 (**Supplementary Table S4**). These findings strongly suggest that the ORF34-encoded protein functions as a depolymerase in phage HHU1.

### 3.6 Phylogenetic analysis of the phage HHU1

**Figure 5:**
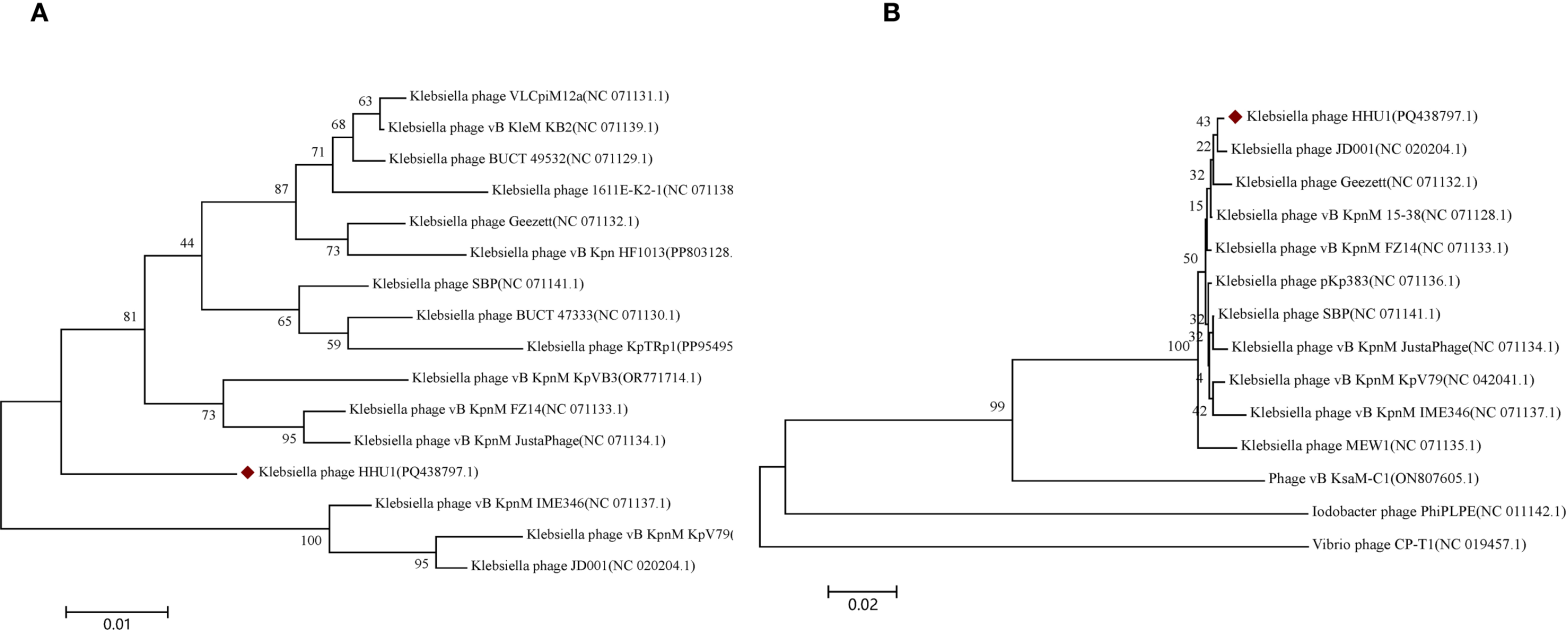
Phylogenetic analysis of phage HHU1. The phylogenetic tree was constructed based on the amino acid sequences of major capsid **(A)** and terminase large subunit **(B)** using the Neighbor-joining method with 1000 bootstrap replicates in MEGA 7. The amino acid sequences of all phages were downloaded from NCBI. Phage HHU1 is labeled with a red rhombus.

To elucidate the evolutionary relationship between phage HHU1 and other phages, phylogenetic trees were constructed using the evolutionarily significant and conserved major capsid protein (ORF63) and terminase large subunit (ORF77) of phage HHU1, aligned with homologous protein sequences obtained from NCBI. In the phylogenetic tree derived from the major capsid protein amino acid sequences, phage HHU1 formed a distinct clade (**Figure 5A**). In contrast, the tree constructed using the large terminase subunit amino acid sequences showed that phage HHU1 clustered with *Klebsiella* phage JD001 (classified within the genus *Jedunavirus*), indicating a close evolutionary relationship between the two phages (**Figure 5B**). Based on these results and the general guidelines established by the International Committee on Taxonomy of Viruses (ICTV) (Turner et al., 2023), phage HHU1 should be classified as a novel species within the genus *Jedunavirus*.

### 3.7 Antimicrobial activity of phage HHU1 *in vitro*

**Figure 6:**
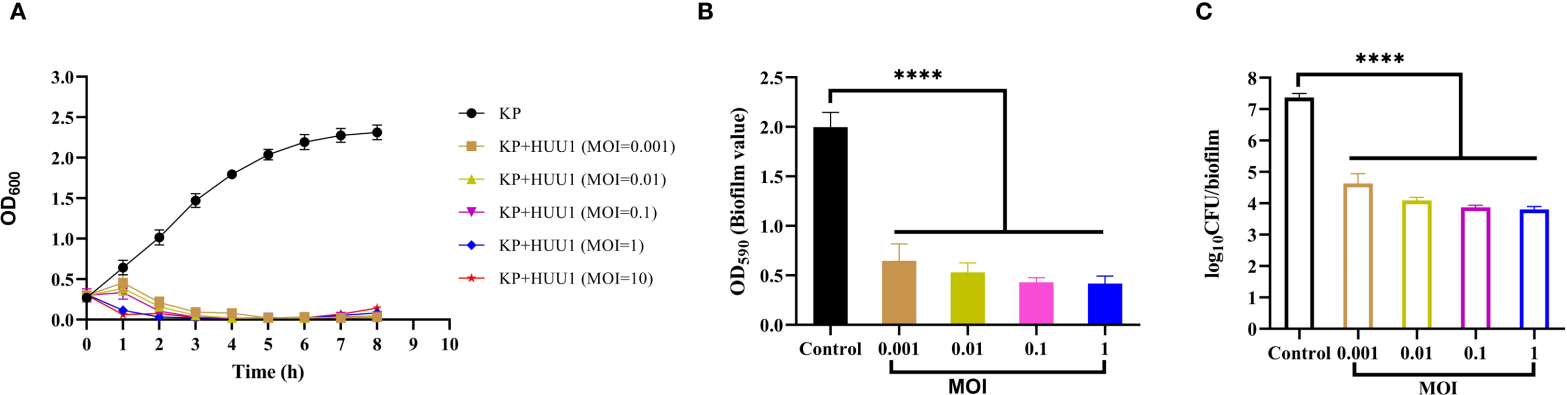
*In vitro* antimicrobial activity of phage HHU1. **(A)** Bacterial growth curves under various MOIs. **(B)** Biofilm formation inhibition efficacy of phage HHU1 against *K. pneumoniae* 1301 at different MOIs. **(C)** Determination of viable cells in biofilms after phage HHU1 treatment with different MOIs. The MOI was calculated using the initial colony-forming units (CFU) of the bacterial host. Data are shown as the mean ± SD. Statistical analysis was performed by one-way analysis of variance following a Dunnett’s multiple comparisons test, ∗∗∗∗*P*< 0.0001.

Using uninfected strain 1301 as the control, the lytic activity of phage HHU1 against strain 1301 was determined at different MOIs. In the control group, bacterial cell density (measured as OD_600_) gradually increased during the experimental period and reached the stationary phase after 7 h of cultivation. In contrast, a significant decrease in absorbance was observed in phage HHU1-infected bacteria at MOIs of 0.001, 0.01, 0.1, 1, and 10. As shown in **Figure 6A**, higher MOIs (ranging from 1 to 10) induced a rapid decline in OD_600_ within less than 1 h. Although a slight increase in OD_600_ occurred within 1 h post-treatment at lower MOIs (ranging from 0.001 to 0.1), a rapid subsequent decrease in OD_600_ was observed over time. In all phage-treated groups, host cell growth was markedly suppressed within 7 h, indicating that phage HHU1 exhibits strong bactericidal activity against strain 1301 *in vitro*.

A key virulence trait of *K. pneumoniae* is its ability to form biofilms, which enhances resistance to antimicrobial agents and host defenses (Guerra et al., 2022). To assess whether phage HHU1 could disrupt preformed biofilms of *K. pneumoniae* 1301 *in vitro*, the crystal violet assay was employed to quantify biofilm biomass after phage treatment. Compared to the negative control group, biofilm formation by bacteria treated with phage HHU1 at various MOIs (ranging from 0.001 to 1) was significantly reduced (**Figure 6B**). Moreover, phages administered at varying MOIs resulted in significant killing of biofilm-embedded bacteria compared to the control group (**Figure 6C**). These findings indicate that phage HHU1 effectively inhibits biofilm biomass and the number of viable bacteria within the biofilm.

### 3.8 *In vivo* therapeutic evaluation of phage HHU1 in a *G. mellonella* infection model

**Figure 7:**
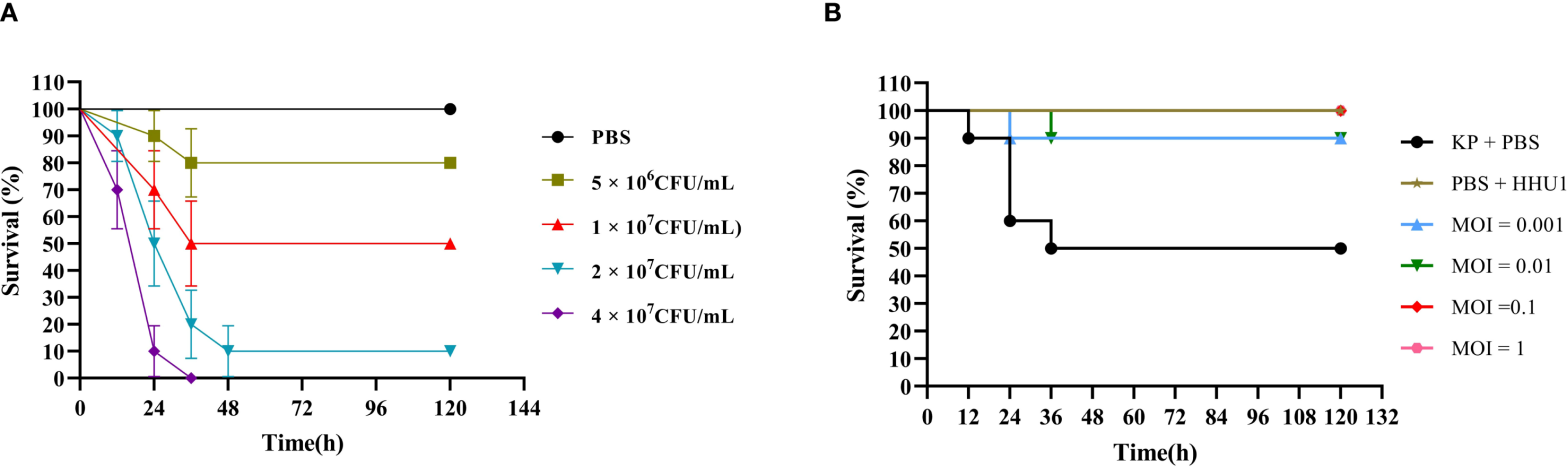
Therapeutic evaluation of phage HHU1 on *Galleria mellonella* model. **(A)** Survival rates of larvae infected with different doses of strain 1301 after 5 days. **(B)** Survival curves of phage HHU1 treatment against larvae infected with strain 1301 after 5 days.

In recent years, the *G. mellonella* model has proven to be an effective tool for evaluating the therapeutic efficacy of phages against bacterial infections (Beeton et al., 2015; Grygorcewicz et al., 2020; Li et al., 2023; Kelly and Jameson, 2024). Therefore, this study utilized *G. mellonella* larvae as an animal model to assess the efficacy of phage HHU1 as an antimicrobial agent for treating infections caused by drug-resistant *K. pneumoniae*. First, the survival of larvae injected with varying concentrations of *K. pneumoniae* strain 1301 was monitored over five days to determine the LD50 of strain 1301 for *G. mellonella* larvae. As shown in **Figure 7A**, a concentration-dependent mortality was observed in larvae post-infection. High bacterial concentrations (4 × 10^7^ CFU/mL) induced 100% mortality within 36 h, while low concentrations (5 × 10^6^ CFU/mL) resulted in only 20% mortality by 120 h. Based on the survival curve, the optimal dose of strain 1301 required to achieve an LD50 in larvae was 1 × 10^7^ CFU/mL.

Subsequently, under the LD50 infection dose, the therapeutic efficacy of phage HHU1 was evaluated by injecting varying concentrations (MOIs ranging from 0.001 to 1). Compared to the bacteria-only control group (50% survival), phage-treated larvae exhibited 100% survival at MOIs of 0.1 and 1, and 90% survival at MOIs of 0.001 and 0.01 (**Figure 7B**). Notably, larvae injected with a high concentration of phage HHU1 (1 × 10^8^ PFU/mL) in the absence of bacterial infection maintained 100% survival, indicating the safety of phage HHU1 for the larvae. These findings demonstrate that phage HHU1 effectively reduces infection caused by drug-resistant *K. pneumoniae* when administered as a therapeutic agent in this *in vivo* model.

## 4 Discussion

Clinical isolates of *K. pneumoniae* are evolving towards increasingly high levels of antimicrobial resistance, establishing this species as one of the most challenging infectious bacterial pathogens to control (Bialek-Davenet et al., 2014). The escalating threat of antibiotic resistance to healthcare systems has led to a diminished efficacy of antimicrobial agents in effectively combating infections, concurrently driving rises in morbidity and mortality (Meybodi et al., 2021). Consequently, the development of novel antimicrobial agents has become a major focus. As natural predators of bacteria, phages exhibit a bactericidal mechanism entirely distinct from that of antibiotics. Capable of killing multidrug-resistant bacteria, phages have emerged as a promising novel therapeutic approach for combating resistant bacterial infections (Peng et al., 2025; Yao et al., 2025). The isolation of lytic phages constitutes the initial step in phage therapy applications. Given that hospital sewage environments harbor mixed populations of various bacteria, phages, antimicrobial agents, and nutrients, they represent a rich reservoir for isolating phages capable of lysing clinically drug-resistant bacteria (Wang et al., 2024; Masalane et al., 2025; Mirza et al., 2025). In this study, a lytic phage (designated as HHU1) targeting multidrug-resistant *K. pneumoniae* was successfully isolated from hospital sewage, and its efficacy was systematically evaluated.

Most *K. pneumoniae* strains are encapsulated by capsular polysaccharides (CPS), which constitute a critical virulence factor promoting bacterial survival during infection and providing pathogen protection (Xu et al., 2024). Phage HHU1 forms plaques with characteristic halos on double-layer agar plates containing host bacteria (**Figure 1A**), This halo phenomenon has been confirmed to result from the activity of a depolymerase, an enzyme produced during phage-mediated lysis that degrades bacterial CPS (Li et al., 2022). Bioinformatic analysis identified that the protein encoded by ORF34 in the HHU1 genome exhibits 99.68% sequence similarity to depolymerase KP32gp38 (Squeglia et al., 2020) from *Klebsiella* phage KP32, further supporting the presence of depolymerase activity in phage HHU1. Depolymerases recognize and degrade bacterial CPS, thereby enabling phages to access and bind secondary receptors on the bacterial surface (Pires et al., 2016). In addition, CPS disruption exposes bacteria to host immune surveillance, facilitating immune clearance and reducing infection severity (Majkowska-Skrobek et al., 2018). Phages encoding depolymerases demonstrate enhanced therapeutic potential due to their dual antibacterial mechanisms.

Biofilms are complex structured communities composed of microbial-derived extracellular polysaccharide matrices, contributing to nearly 80% of bacterial infections (Singh et al., 2019). These structures protect bacteria against host defense mechanisms and the action of antibiotics. Biofilm formation by *K. pneumoniae* represents a critical step in its pathogenesis, facilitating colonization of the respiratory, gastrointestinal, and urinary tracts, and contributing to the development of invasive infections (Wang et al., 2020). Consequently, effective biofilm eradication constitutes a major research focus in *K. pneumoniae* infection treatment. Recent studies reveal that depolymerases not only facilitate the degradation of CPS but also disrupt biofilm formation and reduce bacterial virulence, highlighting their critical roles in phage infection dynamics and host-pathogen interactions (Wu et al., 2019; Dunstan et al., 2023). In this study, phage HHU1 effectively inhibited biofilm formation by clinical *K. pneumoniae* isolates, exhibiting an MOI-dependent manner where higher MOIs correlated with enhanced inhibitory activity (**Figure 6B**). This observation aligns with findings previously reported for *K. pneumoniae* phages Kpph9 (Huang et al., 2025) and P560 (Li et al., 2022), which also possess depolymerase activity. Collectively, these results suggest that phages encoding depolymerases hold substantial potential for preventing and treating biofilm-associated *K. pneumoniae* infections.

Phages initiate infection by recognizing and binding to specific cell surface structures, with CPS serving as both common receptors and critical determinants of phage host range specificity (Latka et al., 2021; Volozhantsev et al., 2022). Host range analysis revealed that phage HHU1 lysed only 7 out of 33 clinically isolated *K. pneumoniae* strains, all belonging to the K2 capsular serotype (**Table 1**). This observation suggests that phage HHU1 may recognize the K2 capsule to initiate infection. This strict host specificity parallels that observed in previously characterized lytic phages targeting K2-*K. pneumoniae*, such as Kpph9 (Huang et al., 2025), B1 (Pertics et al., 2021), and vB_KlebPS_265 (Yakubovskij et al., 2025). Traditional serotyping methods classify *K. pneumoniae* into at least 79 chemically distinct serotypes based on variations in capsule composition and structure (Pan et al., 2015). Even a single broad-spectrum phage is insufficient to target a substantial proportion of all described capsular types. To overcome the challenge of the narrow host range inherent to individual phages, formulating a phage cocktail comprising phages recognizing distinct *K. pneumoniae* capsular types presents a viable strategy. Phage cocktail therapy has been demonstrated to achieve enhanced therapeutic efficacy (Chen et al., 2024; Rotman et al., 2024).

For practical applications, phages must withstand diverse environmental conditions, as their stability under varying temperatures and pH levels directly impacts their therapeutic efficacy against pathogens. In this study, phage HHU1 demonstrated considerable stability across a broad temperature range (4–60°C) and pH spectrum (pH 5–10), showing comparable environmental resistance to previously characterized *K. pneumoniae* phages vB_KpP_HS106 (Chen et al., 2023) and vB_KlebPS_265 (Yakubovskij et al., 2025). The superior stability of HHU1 holds significant value for the future development of phage-based formulations suitable for oral, topical, or systemic administration. Previous studies have established that phages with large burst sizes and short latent periods correlate with enhanced bacterial inactivation efficiency (Abedon et al., 2001). The one-step growth curve analysis of HHU1 revealed a latent period of 10 min and a burst size of approximately 134 PFU/cell. These kinetic parameters surpass those reported for *K. pneumoniae* phages vB_KlebPS_265 (15 min, 26 PFU/cell) (Yakubovskij et al., 2025) and Kpph9 (11 min, 25 PFU/cell) (Huang et al., 2025), indicating superior lytic performance.

Sequencing and analysis of phage genomes enhance the understanding of phage genomics and improve the reliability and safety of phages for clinical therapeutic applications. Therapeutic phages should avoid carrying genetic features that may exacerbate infection risks, such as lysogeny-associated genes, toxin genes, antibiotic resistance genes, and genes encoding highly immunogenic proteins (Skurnik et al., 2025). Notably, no genes linked to antibiotic resistance, virulence, or lysogeny were detected in phage HHU1, indicating its lytic nature and safety profile—essential prerequisites for its future clinical deployment.

Assessment of phage antimicrobial activity *in vitro* can be achieved by measuring bacterial growth kinetics in the presence of phages. In this study, phage HHU1 completely suppressed the growth of its host bacterium within the 8 h testing period across all MOIs (**Figure 6A**), exhibiting a prolonged suppression duration compared to *K. pneumoniae* phages vB_KlebPS_265 (Yakubovskij et al., 2025) and Kpph9 (Huang et al., 2025). The *G. mellonella* model, valued for its low cost, ease of implementation, and possession of an innate immune system highly analogous to humans, has been widely employed to investigate pathogen virulence and the therapeutic efficacy of phages against bacterial infections (Beeton et al., 2015; Kling, 2020; Kelly and Jameson, 2024). In a LD_50_ infection model using *G. mellonella*, treatment with phage HHU1 at the high MOIs (MOI = 0.1 and 1) achieved a 100% survival rate (**Figure 7B**). This survival rate exceeds those previously reported for *K. pneumoniae* phages vB_KpnP_KL106-ULIP47 (Thiry et al., 2019) and vB_KpnS_SXFY507 (Feng et al., 2023) in treating infected *G. mellonella*. This study administered phage therapy at 2 h post-infection in *G. mellonella*. The effects of prophylactic phage therapy (administered prior to bacterial infection) or co-injection therapy (simultaneous administration of bacteria and phage) were not evaluated. However, previous studies have demonstrated that prophylactic phage therapy and co-injection phage therapy are more effective than remedial phage therapy in improving survival rates of *K. pneumoniae*-infected *G. mellonella* larvae (Kelly and Jameson, 2024). The favorable antimicrobial efficacy of phage HHU1 against *K. pneumoniae* both *in vitro* and *in vivo* presents opportunities for its future application as an antimicrobial agent to control infections caused by drug-resistant *K. pneumoniae.* This study did not assess the frequency of resistant mutant emergence during phage treatment. This factor represents a critical determinant of long-term therapeutic efficacy and warrants further investigation in future studies.

## Data Availability

The original contributions presented in the study are publicly available. This data can be found here: https://www.ncbi.nlm.nih.gov/nuccore/2843366331.
