# Supplementary material for: Isolation, characterization and therapeutic evaluation of phage HHU1 against K2 Klebsiella pneumoniae
Source: Front Cell Infect Microbiol. 2025 Sep 30;15:1668727. doi: 10.3389/fcimb.2025.1668727 (PMC12518310; doi:10.3389/fcimb.2025.1668727)
Supplement: Supplementary file 1 [file Table1.docx]

Supplementary Table 1 Minimum inhibitory concentration (MIC) results of antibiotics against K2-type *Klebsiella pneumoniae* strains

| Antibiotics | 1301 | 1307 | CH-03 | CH-05 | CH-71 | CH-113 | CH-188 |
| --- | --- | --- | --- | --- | --- | --- | --- |
| Piperacillin/Tazobactam | >=128 | 64 | >=128 | >=128 | 32 | <=4 | >=128 |
| Ceftazidime | >=64 | 32 | >=64 | >=64 | 4 | <=0.25 | >=64 |
| Cefoperazone | >=64 | 32 | >=64 | >=64 | 32 | <=8 | >=64 |
| Cefepime | >=32 | 4 | >=32 | >=32 | 2 | <=0.25 | >=32 |
| Aztreonam | >=64 | <=1 | >=64 | >=64 | 2 | <=1 | >=64 |
| Imipenem | >=16 | <=0.25 | <=0.25 | 1 | <=0.25 | <=0.25 | 4 |
| Meropenem | >=16 | 0.5 | 0.5 | <=0.25 | <=0.25 | <=0.25 | 1 |
| Amikacin | >=64 | <=2 | 4 | >=64 | <=2 | <=2 | 4 |
| Tobramycin | >=16 | <=1 | >=16 | >=16 | <=1 | <=1 | >=16 |
| Ciprofloxacin | >=4 | <=0.25 | >=4 | <=0.25 | <=0.25 | <=0.25 | <=0.25 |
| Levofloxacin | >=8 | <=0.25 | >=8 | 1 | 1 | <=0.12 | >=8 |
| Doxycycline | 4 | 1 | >=16 | >=16 | 1 | 1 | >=16 |
| Minocycline | 4 | <=1 | 8 | >=16 | <=1 | <=1 | 8 |
| Tigecycline | 2 | <=0.5 | 1 | >=8 | <=0.5 | <=0.5 | 1 |
| Colistin | <=0.5 | <=0.5 | <=0.5 | <=0.5 | <=0.5 | <=0.5 | <=0.5 |
| Trimethoprim-sulfamethoxazole | 40 | <=20 | <=20 | 80 | >=320 | <=20 | <=20 |

Supplementary Table 2 The lytic activity of phage HHU1 against the tested bacterial strains.

| **Species** | **Strains** | **Susceptibility** | **Origin** |
| --- | --- | --- | --- |
| *Pseudomonas aeruginosa* | 4877 | - | 301 Hospital |
|  | 4878 | - | 301 Hospital |
|  | 4879 | - | 301 Hospital |
|  | 4880 | - | 301 Hospital |
| *Acinetobacter baumannii* | 3288 | - | 306 Hospital |
|  | 3289 | - | 306 Hospital |
|  | 3290 | - | 306 Hospital |
|  | 3291 | - | 306 Hospital |
| *Escherichia coli* | 3251 | - | 306 Hospital |
|  | 3252 | - | 306 Hospital |
|  | 3253 | - | 306 Hospital |
|  | 3254 | - | 306 Hospital |
| *Stenotrophomonas maltophilia* | 118 | - | 307 Hospital |
|  | 209 | - | 307 Hospital |
|  | 532 | - | 307 Hospital |
|  | 548 | - | 307 Hospital |

Symbols: "-" indicates no plaque formation following co-culture of phage HHU1 with the test bacterium.

Supplementary table 3 Analysis of phage HHU1 genome-encoded proteins using DePP

| ORFs | Function | Probability_DePol |
| --- | --- | --- |
| ORF34 | tail protein | 0.929261111 |
| ORF8 | DNA primase | 0.813202116 |
| ORF39 | baseplate hub | 0.789177513 |
| ORF23 | DNA polymerase | 0.760813492 |
| ORF40 | tail length tape measure protein | 0.748828307 |
| ORF62 | endonuclease-like protein | 0.701662698 |
| ORF53 | tail sheath | 0.673404233 |
| ORF76 | portal protein | 0.663711111 |
| ORF35 | baseplate wedge subunit | 0.638726984 |
| ORF63 | major head protein | 0.594572487 |
| ORF12 | helicase | 0.560541799 |
| ORF77 | terminase large subunit | 0.547180159 |
| ORF18 | exonuclease | 0.522392593 |

Note: Only ORF-encoded proteins with probability values greater than 0.5 are listed in the table

Supplementary table 4 Comparison results of ORF34-encoded proteins with PDB proteins

| ORF | Residues | Hit | PDB ID | Probability (%) | E-value |
| --- | --- | --- | --- | --- | --- |
| 34 | 144-677 | Depolymerase KP32gp38 | 6TKU_A | 99.68 | 4.1e-13 |
